# A Severe Case of Odontogenic Infection and Necrotizing Fasciitis of the Anterior Chest Wall and Neck

**DOI:** 10.7759/cureus.22438

**Published:** 2022-02-21

**Authors:** Mallorie L Huff, Kyle S Wilson, Kathleen E Kane, Kathryn L Wheel, Joseph J Stirparo

**Affiliations:** 1 Department of Surgery, Division of Trauma and Acute Care Surgery, University of South Florida Morsani College of Medicine/Lehigh Valley Health Network Campus, Bethlehem, USA; 2 Department of Emergency and Hospital Medicine, University of South Florida Morsani College of Medicine/Lehigh Valley Health Network Campus, Bethlehem, USA

**Keywords:** thoracic wall, thoracic extension, polymicrobial, necrotizing fasciitis, dental focal infection, craniocervical

## Abstract

Necrotizing fasciitis is a life-threatening infection that can be rapidly fatal. Early identification and emergent surgical management are essential to minimize morbidity and mortality. This case report describes a 25-year-old male who presented to the emergency department with a three-day history of worsening left lower dental infection and new-onset neck pain and swelling. He received broad-spectrum antibiotics and intravenous fluid resuscitation and underwent computed tomography of the neck and chest. Following intensive care unit admission, he underwent tooth extraction where intraoperative evaluation revealed subcutaneous crepitus. Immediate debridement was performed, revealing copious foul-smelling purulent discharge and necrotic tissue extending over the anterior chest wall and neck. During his hospital course, he underwent multiple debridements to manage the expanding infection. The final tissue defect was substantial, with deep dissection to muscle extending over the entire anterior surface of the rib cage to just inferior to the clavicles. This significant tissue defect was managed with skin grafts, and he was discharged home in stable condition. The patient is doing well almost a year after discharge. The key to our patient’s survival was the early identification and debridement of the affected tissue. Our study reinforces the tenants of wound care and aggressive management required to bolster patient odds of survival in the setting of necrotizing fasciitis and underscores the importance of maintaining vigilance in patients presenting with dental infections. This study is unique in that our patient was young, with a past medical history significant for polydrug use, and the area of debridement was substantial.

## Introduction

Necrotizing fasciitis is a rare, life-threatening infection that can become rapidly fatal, with an estimated annual incidence of approximately four in 100,000 [1]. Patients who develop necrotizing fasciitis are typically older, immunocompromised individuals [2]. Diabetes mellitus is the most common predisposing factor and predictor of mortality when dealing with necrotizing fasciitis [2]. The mortality rate for necrotizing fasciitis is approximately 25 percent, increasing to a 50 percent mortality rate with intensive care unit (ICU) admission [1]. However, without surgical intervention, the mortality rate is nearly 100 percent [3].

Necrotizing fasciitis most commonly occurs in the extremities, abdomen, perineum, and buttocks. Craniocervical necrotizing fasciitis (CCNF) is rare, composing 1 to 10 percent of reported cases [3]. The most common etiology of CCNF is an odontogenic infection following a recent dental procedure, maxillofacial trauma, or hygiene neglect. Odontogenic infections are often polymicrobial, include local mouth and skin flora, and are notoriously aggressive [2]. CCNF typically extends inferiorly via anatomical conduits including the carotid sheath, paratracheal fascia, retropharyngeal space, or prevertebral fascia. Thoracic extension of CCNF occurs in 50 percent of CCNF patients and is associated with a 40 percent increase in mortality [4,5].

## Case presentation

**Figure 1:**
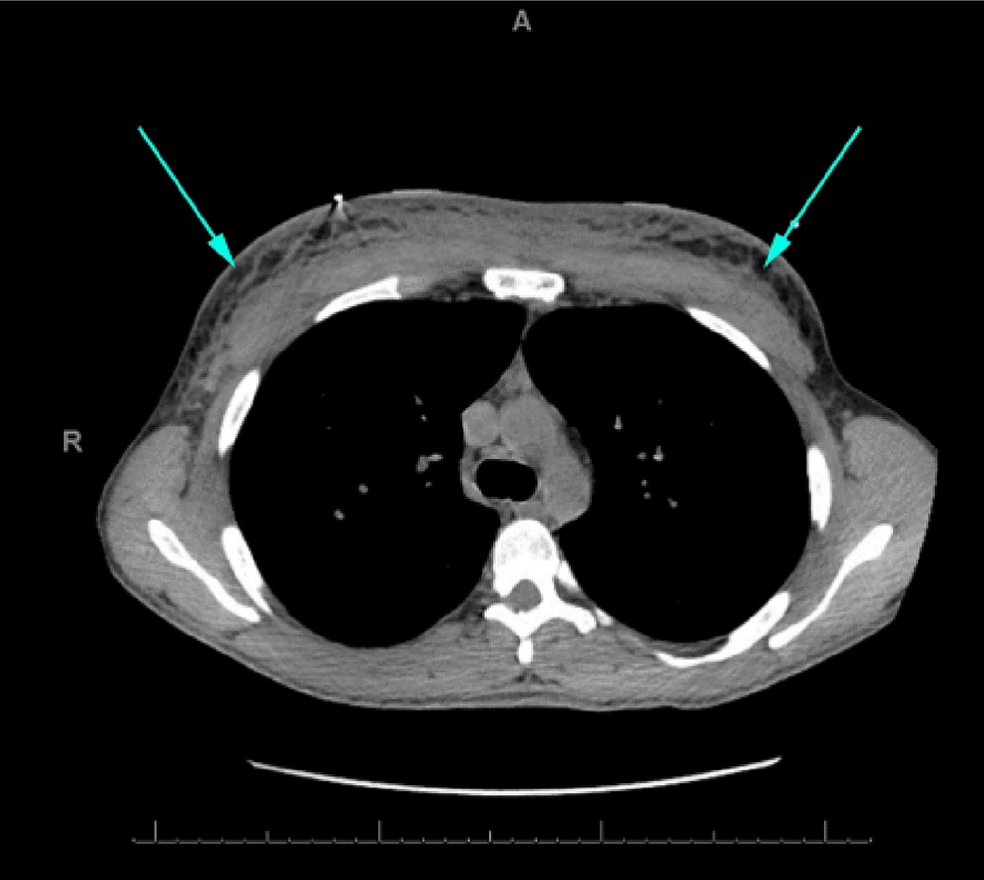
Computed tomography of the chest on admission without contrast. Axial view showing significant diffuse body wall edema with reticulation of subcutaneous tissue (arrows).

**Figure 2:**
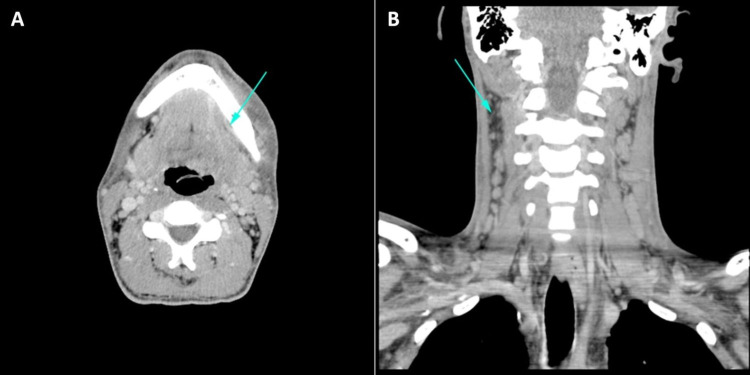
Computed tomography of the head and neck on admission with intravenous contrast. Axial section (A) showing a small contrast-enhancing mass consistent with abscess (arrow). Coronal section (B) showing extensive reticulation within the subcutaneous soft tissues, reflecting diffuse cellulitis (arrow).

A 25-year-old man with a history significant for tobacco, marijuana, and methamphetamine use presented to the emergency department with a three-day history of worsening left lower dental infection in the setting of a broken molar with a new-onset neck and chest swelling and redness. In the emergency department (ED), he was found to be febrile (38.5^o^C) and tachycardic (126 BPM) with a leukocytosis of 13.8 thou/cmm (4.5-11) and elevated serum lactate of 5.7 mmol/L (0.5-2.2). His other vitals included a blood pressure of 110/67 mmHg, respiration 19 BrPM, and oxygen saturation of 93% on room air. A chest computed tomography (CT) revealed evidence of diffuse cellulitis with significant body wall edema most prominent in the anterior superior chest and neck (Figures 1-2). He was initiated on ampicillin/sulbactam and vancomycin and admitted to the ICU for resuscitation and airway monitoring out of concern for eminent airway compromise. Initial resuscitation with intravenous fluids resulted in adequate urine output and net five liters positive prior to surgery.

**Figure 3:**
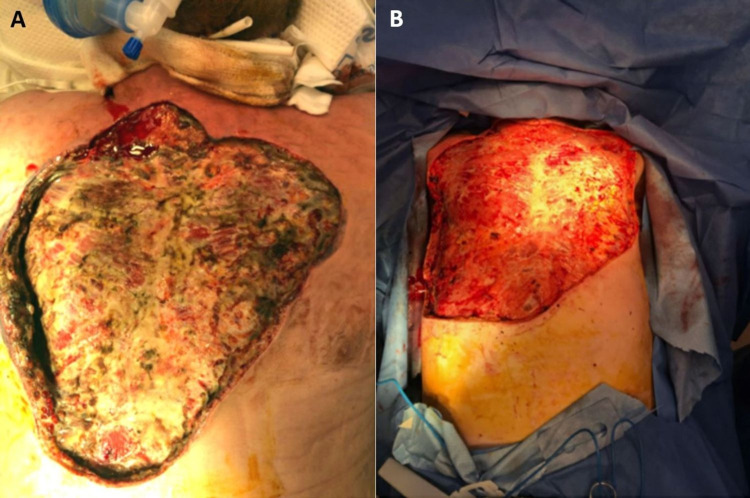
Debridement area as documented 1-day status post first debridement, prior to second-look surgery (A) and immediately following the final debridement (B). Disclaimer: Photoshop was used to remove unique skin markings to preserve patient confidentiality, but the wound beds were not digitally altered.

An oral surgery consult identified a dental abscess with draining purulence distal to tooth number 19, noting that the left submandibular space was firm to touch. Incision and drainage of the left and right submandibular space and submental space were performed with the extraction of teeth 16, 18, and 19. Copious purulent discharge was expressed and sent for cultures. Quarter-inch Penrose drains were placed in the bilateral submandibular spaces, the submental space, and between the bilateral submandibular spaces and the submental space. Intraoperatively, subcutaneous crepitus was appreciated over the neck and left anterior chest. An incision was made, revealing significant foul-smelling purulent discharge and liquidated necrotic tissue, confirming the diagnosis of necrotizing fasciitis. Trauma surgery was immediately contacted for intraoperative consultation as they were already consulted on the case given the potential for airway compromise. The chest incision over the left anterior chest wall was deepened to the level of fascia and opened widely, debriding all necrotic fascia and overlying tissue until healthy bleeding tissue was visible. The wound was packed with a betadine-soaked gauze dressing and dry dressings with a plan for second-look surgery the following day (Figure 3A).

Following the initial debridement, he returned to the ICU where he remained sedated with an endotracheal tube and ventilator support as his condition stabilized. The endotracheal tube was later replaced with tracheostomy tube. Wound cultures revealed a polymicrobial infection with aerobes and anaerobes including viridansstreptococci (*Streptococcus anginosus*), *Cutibacterium* *acnes *(*propionibacterium*), and *Peptostreptococcus asaccharolyticus*. The infectious disease service was consulted for antibiotic management, and he was placed on ampicillin-sulbactam and vancomycin as guided by culture sensitivity. He returned to the operating room seven times for additional debridement and washout of his chest wall with temporizing vac-assisted closure with negative pressure wound therapy (NPWT) to promote wound drainage and granulation tissue formation. His postoperative course was complicated by hematoma formation, requiring operative intervention, and anemia requiring blood transfusion. The patient was found to be HIV-negative.

After 18 days of NPWT with excellent granulation tissue formation, split-thickness skin grafts (STSG) were harvested from his bilateral lower legs and adhered with staples to the chest wall. In total, the repair required 46 cm by 50 cm of graft for repair (Figure 3). A wound vac was placed and maintained for seven additional days; and, upon removal, 90 percent of the graft was adherent. Ultimately, the patient was discharged home with a wound vac to be maintained with home health visits. Now, a year from discharge, he has fully healed and is doing well.

## Discussion

There are significant challenges in managing patients with CCNF, especially because early identification and treatment are key for improved prognostication. As the initial stages of necrotizing fasciitis are nonspecific and overt signs of sepsis are not present, identification in a medical setting may not occur until the disease has progressed significantly [5]. Misdiagnosis or delayed diagnosis is common and leads to ineffective resource use as well as a worse prognosis [1].

It is universally agreed that prompt, aggressive surgical debridement with removal of all affected or at-risk tissue and initiation of broad-spectrum antibiotics are essential in improving patient outcomes [1,2,5]. It is also estimated that up to 80 percent of patients who died as a direct result of necrotizing fasciitis had significant delays in diagnosis and operative intervention [6]. The most important factor in the management of patients with necrotizing fasciitis is immediate aggressive surgical debridement [7]. High-dose broad-spectrum antibiotics, while an essential component in the management of necrotizing fasciitis, are an adjuvant to surgical debridement. As local coagulation limits tissue perfusion in affected areas, antibiotics alone are insufficient to be an independent cure but can limit the spread of infection to vulnerable tissues.

CCNF offers unique challenges in patient management such as airway obstruction, carotid artery occlusion, jugular vein thrombosis, mediastinitis, pneumonitis, and pleural effusion due to its proximity to vital anatomical structures [4]. There should be a low threshold for intubation when dealing with CCNF, as local swelling can lead to obstruction of the trachea and airway collapse [8]. Further, as serial debridement is often required, early tracheostomy is recommended to prevent damage secondary to long-term endotracheal tube use [3].

Reconstruction after necrotizing fasciitis can be challenging as extensive skin and soft-tissue loss can create massive tissue defects with potentially compromised vascular supply. Further, wound healing may be delayed in the setting of poor nutritional status, and wound complications, including hematoma formation, are common [2,5]. In our patient’s case, NPWT was a highly successful temporizing closure that aided future reconstructive efforts. His wound beds exhibited excellent granulation tissue formation prior to grafting and STSG was highly successful with 90 percent graft uptake on the first attempt. We attribute this noncomplicated reconstruction to consistent use of NPWT throughout the patient’s hospital stay, our patient’s young age, and lack of comorbidities.

## Conclusions

Early identification of necrotizing fasciitis with immediate surgical intervention is essential in improving patient outcomes. This can be challenging since initial symptoms of necrotizing fasciitis are nonspecific. The highly aggressive nature of odontogenic infections and CCNF’s proximity to vital structures in the head and neck further increase the importance of rapid diagnosis and complicate its management. Given that patients with CCNF are typically elderly males with preexisting immunocompromised status, our report of a young man without significant past medical history is highly unusual. Our study reinforces the tenants of rapid diagnosis, aggressive surgical management, and principles of infection control and wound care required to bolster patient odds of survival in the setting of necrotizing fasciitis.
